# Flow Cytometry-Detected Immunological Markers and on Farm Recorded Parameters in Composite Cow Milk as Related to Udder Health Status

**DOI:** 10.3390/vetsci7030114

**Published:** 2020-08-17

**Authors:** Giovanna De Matteis, Francesco Grandoni, Maria Carmela Scatà, Gennaro Catillo, Bianca Moioli, Luca Buttazzoni

**Affiliations:** Research Centre for Animal Production and Aquaculture, Consiglio per la Ricerca in Agricoltura e l’Analisi dell’Economia Agraria (CREA), 00015 Monterotondo (Roma), Italy; francesco.grandoni@crea.gov.it (F.G.); mariacarmela.scata@crea.gov.it (M.C.S.); gennaro.catillo@crea.gov.it (G.C.); lcapril82@gmail.com (B.M.); luca.buttazzoni@crea.gov.it (L.B.)

**Keywords:** flow cytometry, milk somatic cells, cell viability, electrical conductivity, milk flow rate

## Abstract

Flow cytometry is a powerful technology used in many fields of cell biology. It is also used as a routine method to count somatic cells in milk and to characterize bovine milk leukocytes. In this study, we used flow cytometry to simultaneously assess the viability, the percentage of the single subsets of leukocytes and to quantify the expression of CD11b, an immunological marker of cell activation status. Immunological markers were then related with on farm recorded parameters as milk electrical conductivity (MEC) and average milk flow rate (MFR). Composite milk samples were collected from 43 cows, nine of which had naturally infected udders and 34 of which had no infected udders. First, the milk samples were classified according to bacteriological test in positive and negative. The results showed that the negative samples to bacteriological test had: (i) significantly higher percentages of live lymphocytes; (ii) significantly lower percentages of CD11b^+^ leukocytes; (iii) significantly lower MEC and higher MFR values. Then, samples were classified in three groups according to somatic cell count (SCC): Group A (*n* = 15), samples with SCC ≤ 100,000 cells/mL, all negative to bacteriological analysis; Group B (*n* = 11), samples with 100,000 < SCC < 300,000 cells/m, with four samples positive to bacteriological analysis; Group C (*n* = 17), samples with SCC ≥ 300,000 cell/mL with five samples positive to bacteriological analysis. Multivariate discriminant analysis was used to verify which flow cytometry immunological markers and on farm recorded parameters could better discriminate among the different groups of SCCs. Linear discriminant analysis (LDA) indicated that 5 of the 10 parameters could best be used to reveal the differences between positive and negative samples among the considered groups of SCCs. Furthermore, the Canonical discriminant analysis (CDA) indicated that composite milk samples with different SCC and infection status were clearly separated from each other in a two-dimensional space. In conclusion, the study highlighted that: (1) the conventional flow cytometry analysis applied on milk samples is a useful tool to investigate immunological parameters as potential indicators of udder health; (2) the combined evaluation of live milk leukocytes and recorded farm parameters could be useful to assess udder health status in dairy cows. The results obtained from this pilot study on few data require new and larger trials to be confirmed.

## 1. Introduction

Somatic cell count (SCC) has been used for a long time as an indicator of udder health and it is an important parameter in the dairy industry since it affects the price of milk. However, because SCC parameter is temporally and individually variable during lactation, it is not always a clear indicator of a potential infection. It has already been demonstrated that the immune cells and in particular the neutrophils, play an important role in the immunity of mammary gland, for the effective defense against invading pathogens and the resolution of infection disease [1,2].

In recent years, besides the determination of SCC, flow cytometry was used to characterize the relative proportion of lymphocytes, macrophages and polymorphonuclear leukocytes (PMN), called differential cell count (DCC) and was proposed as a valid tool to identify inflammatory processes in animals with low SCC [3,4]. Differential somatic cell count (DSCC), a method for routine mastitis screening by a milk analyzer has been described by Damm and coworkers [5]. More recently, Wall and colleagues [6] indicated that the combination of DSCC and SCC could lead to increased sensitivity in mastitis monitoring. Furthermore, a newly published study showed that the DSCC may be a marker to identify early changes in milk composition as a result of an alteration in the milk secretion mechanism [7].

The immunity of mammary gland is of great importance for the resolution of inflammatory and infectious processes in dairy cows and the study of its components is of major interest to understand the infection control mechanisms. In this study, we wanted to investigate during an infection, in addition to the dynamics of the various cell populations also the expression of β-integrin CD11b. The expression of this molecule allows the activation of phagocytes (PMN and macrophages) during infection [8]. It also seems to be associated with T cell activation [9,10] and thought to be a homing receptor of T lymphocytes for infected or inflamed sites [11].

Moreover, the precision livestock farming (PLF) that uses tools for real-time monitoring of livestock, is increasingly developing, thus allowing to optimize production efficiency and animal welfare. PLF focuses on improving the life of animals by warning the farmer when problems arise, so enabling him to take immediate actions [12]. Multiple automated data collection systems based on sensors have been tested to detect mastitis from milk data (e.g., daily milk weights, milk composition, electrical conductivity, SCC, rumination and activity) [13]. In order to evaluate both immune and functional udder parameters, we have chosen to use composite milk samples. Previous studies evaluated the suitability to use composite milk samples to detect intramammary infection in dairy cow: these samples may be useful when considering management options at the cow and herd level and they are more economical to use in culturing protocols than individual quarter samples [14,15].

To our knowledge, this is the first study that evaluate immunological parameters as milk leukocytes subsets and CD11b expression by conventional flow cytometer and on farm collected data such as milk electrical conductivity (MEC) and average milk flow rate (MFR) in composite milk samples.

The aims of this study were: (i) the assessment of a polychromatic flow cytometric assay to evaluate each subset of milk somatic cells; (ii) the investigation of CD11b expression on membrane of milk leukocytes; (iii) the comparison of flow cytometry-detected parameters with automated recorded data: MEC and MFR; (iv) finally, to test whether considered parameters could be used to assign milk samples by categories of microbiological status and SCC.

## 2. Materials and Methods

### 2.1. Animals and Milk Samples Collection

The management and care of experimental animals was carried out in compliance with the 2010/63/EU directive. Italian Holstein lactating cows were kept at the Research Center for Animal Production and Aquaculture of CREA located in center of Italy. Cows were milked in a milking parlor with automatic milk recorders (DeLaval International AB, Kansas City, MO, USA) and data were recorded and stored by the AlProHerd management system software (DeLaval). 

For this trial, composite milk samples were collected manually during routine management controls from 43 cows, aged from 2nd to 4th lactation within two weeks from calving. The milk samples were collected during the morning milking procedure, after accurate cleaning the teats and eliminating the first jets. Milk was collected in sterile tube by taking an equal amount of milk from all four quarters.

Aliquots of milk samples were immediately transported under refrigerated conditions to an external laboratory (Istituto Zooprofilattico Sperimentale Lazio e Toscana, IZSLT, Rome, Italy) for routine analysis (somatic cell count and bacteriological analysis). The somatic cell enumeration was performed with the routine method operating with a fluoro–opto–electronic cell counter and the bacteriological analysis were performed by traditional bacterial culture for the identification of bovine mastitis bacteria. According to the bacteriological results, milk samples were classified into two groups: POS Group (*n* = 9) and NEG Group (*n* = 34). The following pathogens were detected: *Coagulase-negative staphylococci* (*n* = 5); *Serratia marcescens* (*n* = 4).

### 2.2. Purification of Milk Somatic Cells

Aliquots of 100 mL were used for flow cytometric assay. Briefly, the milk samples were centrifuged at 800× *g* for 20 min at 4 °C and the fat layer and supernatant were discarded. The purified cell pellets were washed twice with cold phosphate buffered saline solution (PBS) and counted with TC10 automated cell counter (Bio-Rad Laboratories, Hercules, CA, USA).

### 2.3. Flow Cytometric Analysis

One-hundred microliters of the cell suspension at 1 × 10^6^ cells/mL were incubated on ice for 30 min in the dark with a flow cytometric four-color panel: 5 µL of FITC-conjugated anti-CD11b (clone CC126, Bio-Rad), 5 µL of PE-conjugated anti-CD45 (clone CC1, Bio-Rad), 5 µL of APC-conjugated anti-CD14 (clone TÜK4, Miltenyi Biotec, Bergisch Gladbach, Germany); 1 µL of the Live/Dead^TM^ Fixable Near-IR Dead Cell (Thermo Fisher Scientific, Waltham, MA, USA) was added to the mix solution and incubated for 10 min in the dark. After incubation, the cells were washed twice with 1 mL of cold PBS and resuspended in 300 μL of PBS for the flow cytometric analysis.

**Figure 1:**
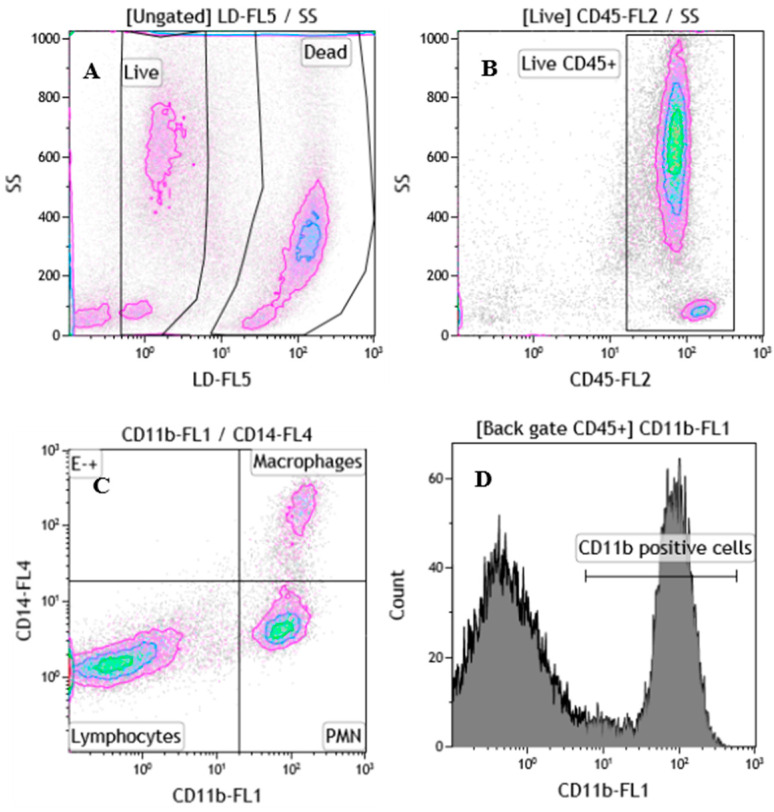
Gating strategy for milk leukocytes subsets identification and CD11b expression. (**A**) Identification of viable milk cells as live/dead negative (LD^−^); (**B**) within live gate, identification of milk leukocytes as CD45^+^ cells; (**C**) identification of live lymphocytes, macrophages and PMN cells; (**D**) histogram showing the CD11b expression on CD45^+^ cells, calculated as mean fluorescent intensity (MFI).

Appropriate gating strategies were used to evaluate live milk leukocytes subsets and CD11b expression (Figure 1). In additions to dead-cell exclusion, live milk cells were identified as live/dead negative (LD^-^) (Figure 1A) and visualized on a dot plot CD45-PE (FL2) vs. side scatter (SS) to select live milk leukocytes as CD45^+^ cells (Figure 1B). PMN and monocytes/macrophages were identified as LD^−^/CD45^+^/CD11b^+^/CD14^−/low^ and LD^−^/CD45^+^/CD11b^+^/CD14^+^ cells, respectively; live lymphocytes were identified by size and granularity (forward-scatter height, FSC-H vs. side-scatter height, SSC-H) and as LD^−^/CD45^+^/CD11b^−/low^/CD14^−^ cells (Figure 1C). At least 10,000 events were collected within each gate. Milk leukocytes expressing CD11b were identified as LD^−^/CD45^+^/CD11b^+^ (Figure 1D). The relative percentage of live leukocytes subsets and the CD11b expression, were evaluated on CytoFlex flow cytometer (Beckman Coulter, Brea, CA, USA) and data were analyzed with Kaluza analysis software v 2.1 (Beckman Coulter).

### 2.4. Statistical Analysis

In order to evaluate the differences between POS and NEG samples, SCC, immunological and automated recorded data were analyzed using the ANOVA procedure of SAS software (SAS Institute, 2012, Inc., Cary, NC, USA) using the following model: Y_ij_ = μ + C_i_ + e_ij_, where: Y_ij_ is the observation vector for the all traits; μ is total average for the traits; C_i_ is the fixed effect of the sample Group POS or NEG (i = 1,2); e_ij_ is the random residual effect.

Furthermore, based on the SCC, samples were divided into 3 groups: Group A (*n* = 15) samples with SCC ≤ 100,000 cells/mL; Group B (*n* = 11) samples with 100,000 < SCC < 300,000 cells/mL; Group C (*n* = 17) samples with SCC ≥ 300,000 cell/mL. Although the optimal SCC cutoff to distinguish between infected and uninfected at the individual cow level was established at 200,000 cells/mL (IDF, 2013), in our trial, composite milk samples were classified according to cellularity as low (Group A), with all samples negative to bacteriological analysis, medium (Group B), with 4 samples positive to bacteriological analysis and high cellularity (Group C), with 5 samples positive to bacteriological analysis. Two analysis procedures were used to examine in a multivariate space the importance of the considered parameters in the definition of POS and NEG groups within the following classes: Class A-NEG, A-POS, B-NEG, B-POS, C-NEG and C-POS. Multivariate discriminant analysis was used to relate the flow cytometry immunological markers and recorded farm parameters with the three classes of SCC. The first implemented procedure was a linear discriminant analysis (LDA) to evaluate the error of assigning subjects to classes. After removing cows with missing data, only 39 of them were left for LDA. The second procedure was a canonical discriminant analysis (CDA). The discriminant function is calculated in order to maximize the ratio among between-group variation and within-group variation. Therefore, the equation can differentiate groups of individuals defined by a generalized distance, Mahalanobis D2 [16]. The analysis was carried out using the Proc DISCRIM of SAS software.

## 3. Results

### 3.1. Flow Cytometry-Detected Immunological Markers and on Farm Recorded Parameters in Different Bacteriological Conditions

Descriptive statistics on immunological traits and recorded farm parameters are reported in Table 1.

Differences between the average value of variables (live leukocytes subset, CD11b expression and recorded farm parameters) between POS and NEG groups are presented in Table 2. Flow cytometric analysis showed that NEG group had a significantly higher percentage of live lymphocytes (*p* < 0.04) and a significantly lower percentage of leukocytes expressing CD11b (*p* < 0.03), as compared with the POS group. Furthermore, the NEG group showed significantly lower values of MEC (*p* < 0.01) and higher values of MFR (*p* < 0.03) than the POS group.

### 3.2. Linear and Canonical Discriminant Analysis

Table 3 reports LDA assignment of subjects (milk samples) to classes identified according to microbiological analysis and SCC. No samples were found in Class A-POS. Milk samples within the Class A-NEG, C-NEG and C-POS were correctly assigned, while the classification of samples to the classes B-NEG and B-POS was less accurate with an error rate of 0.67 and 0.5, respectively.

Canonical analysis identified 4 canonical functions (Table 4), with the first two absorbing almost all the variance (88%). However, only the first canonical variate is significant (*p* = 0.007), as confirmed by the low Wilks’ Lambda value. During the variable selection process, each discriminant function was calculated to maximize the variation among classes and to minimize the variation within a class. Consequently, the coefficient of canonical function may be considered to evaluate the relative importance of the parameter in the discrimination. Therefore, the percentages of macrophages, PMN, lymphocytes and MEC were the parameters that had the highest relative weights on the first canonical variate (Can1), while MFR was on the second variate (Can2).

In discriminant analysis, each sample is a point in a m-dimensional space (m being the number of canonical variates). Since the difficulty to visualize the data in m-dimensional space, the discriminant analysis can reduce the dimensions of the system with a marginal loss in total variability. In this case, 88% of the variance is explained by the first two canonical variates and the system may be reduced to two dimensions only. The first canonical variate is a linear combination of parameters that best discriminates among classes and is plotted on the X-axis. The second canonical variate is the next best linear combination and is plotted on the Y axis. Therefore, each sample can be characterized by a pair of canonical variates.

**Figure 2:**
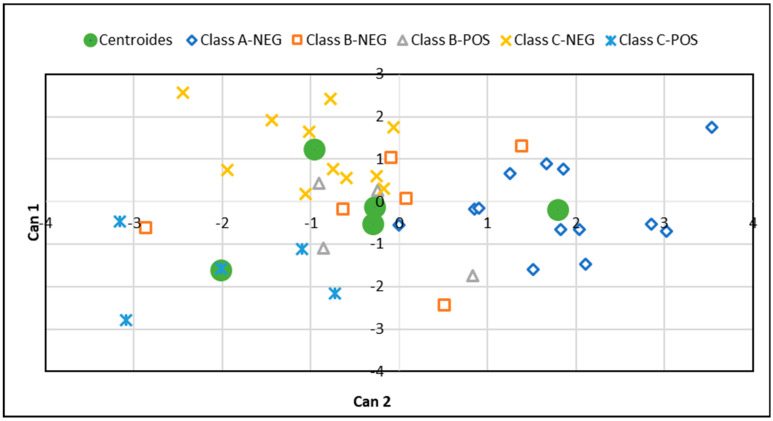
Canonical plot on two dimensions with centroids and individual scores. Canonical variates (Can1 and Can2) were computed from the 5 original variables: percentage of lymphocytes, polymorphonuclear leukocytes (PMN), macrophages and recorded farm parameters milk electrical conductivity (MEC) and average milk flow rate (MFR) selected in the discriminant analysis. The green circles represent the centroids for each class.

Figure 2 graphically represents the canonical analysis in a two-dimensional space. In the graph each sample is plotted according to its canonical variates and centroids represents the mean of the data points in each class. As shown by the plot, Group A-NEG was significantly separated from classes C-NEG and C-POS, with a *p*-value of the Mahalanobis distance of 0.002 and 0.001, respectively (Table 5). Furthermore, classes C-NEG and C-POS were significantly distant from each other, with a *p*-value of the Mahalanobis distance of 0.038 (Table 5). However, classes B-NEG and B-POS were not significantly distant from each other and from the other classes.

## 4. Discussion

Measuring somatic cell count (SCC) is a simple way to monitor udder health on herd level [14]. However, this count does not differentiate into lymphocytes, PMN and macrophages, the principal cell populations present in milk. Several studies reported that the relative proportions of these cells play an important role in the immunity of the mammary gland and the resolution against invading pathogens depends on number and distribution of leukocyte subsets [1,2]. Milk somatic cells from inflamed gland are mostly neutrophils [17] because the inflammation provokes a dramatic shift in the composition of the SCC from mononuclear to PMN cells [18].

The DSCC has recently been described as a new tool for monitoring udder health [5,6] based on the percentage of PMN combined with lymphocytes. Proportions of macrophages can be calculated by 100-DSCC. This analysis is performed by high-speed flow cytometry technology [5].

Wall and coworkers [6] investigated the changes in both DSCC and SCC in udder quarters during mastitis induced by cell wall components from typical mastitis-causing pathogens [lipopolysaccharide (LPS), *Escherichia coli*; and lipoteichoic acid (LTA), *Staphylococcus aureus*]. They showed that higher SCC and DSCC value were seen after the stimuli, indicating that the composition of the immune cells changed after stimulation. Even though DSCC represents the proportion of both PMN and lymphocytes, authors ascribe this change only to the increase in the percentage of PMN, as this is the predominant population observed in inflamed glands, whereas lymphocytes play a minor role as previously discussed by Damm and coworkers [5].

In our study, we used a conventional flow cytometer and we assessed a flow cytometric assay to identify in composite milk samples the relative proportion of each single leukocytes population, taking into account also the cell viability phenotype; in addition, we evaluated the expression of an inflammatory marker as β-integrin CD11b on the surface of leukocytes subsets.

Human studies reported that CD11b immunophenotyping is useful to identify inflammatory profiles [19]. It also seems to be associated with T cell activation [9,10] and thought to be a homing receptor of T lymphocytes for infected or inflamed sites [11]. In bovine, it was shown that the expression of CD11b on circulating neutrophils steadily increased upon intramammarily administration of endotoxin lipopolysaccharide (LPS) [20]. Our results seem in agreement with these studies because CD11b is significantly higher in milk samples positive to bacteriological analysis, indicating inflammatory status. Considering the evidence that in inflammatory conditions the expression of the integrin CD11b increases in all milk leukocytes (CD45^+^ cells), we suppose that the expression of CD11b could be considered as a marker of udder inflammation.

The joint consideration of immunological markers together with recorded farm parameters showed significant associations with udder health status. MFR is one of the milk flow traits that characterize the milkability, an economically important functional trait. Genomic associations studies showed relationship between electronically measured milk flow traits and mammary health indicator traits (SCC, SCS, or clinical mastitis) [21]. MEC is a simple and cheap parameter used as an indicator of mastitis [22]: Vilas Boas and coworkers [23] reported a positive correlation between SCC and MEC. However, the same authors suggested that MEC should be used in association with other milk parameters. In our study, we showed that MEC and the percentage of CD11b positive leukocytes increase in bacteriological positive milk samples than negative. This result agrees also with Alhussien et al. [24] that observed a significant increase of MEC in cows with clinical mastitis and significant increase in mRNA expression of *CD11b* on isolated milk PMN from cows with sub-clinical and clinical mastitis.

Moreover, multivariate discriminant analysis was used to verify which flow cytometry immunological markers and on farm recorded parameters could better discriminate among the different groups of SCCs. LDA indicated that 5 of the 10 parameters could best be used to reveal the differences between positive and negative samples among the considered groups of SCCs. Furthermore, CDA indicated that composite milk samples with different SCC and different infection status have distinct characteristics.

## 5. Conclusions

In this study, a polychromatic flow cytometric assay was assessed to evaluated, in composite milk samples, each subset of milk somatic cells and to investigate the CD11b expression. Results showed that the percentage of live lymphocytes and the expression of CD11b on milk leukocytes could be considered as potential biomarkers of udder health status in dairy cows. Furthermore, the flow cytometry-detected parameters were compared with automated recorded data, MEC and MFR, to test whether considered parameters could be used to classify milk samples by categories of microbiological status and SCC. The multivariate analysis highlighted that live lymphocytes, PMN, macrophages and the MEC could be used together with SCC to assess the health status of cows from composite milk samples.

Overall, the findings of this preliminary study suggest that conventional flow cytometry approach applied on milk samples can be effectively used to identify new immunological markers of udder health status. However, further analysis should be performed to confirm results of this pilot study. Finally, more research on this topic should be increased to assess the functional status of milk leukocytes and to discover new immunological markers and their potential application.

## Figures and Tables

**Table 1:** Descriptive statistics of immunological traits and automated recorded farm parameters.

Item	*n*	Mean	St. Dev	Minimum	Maximum
Cell viability (%)	40	54.70	17.76	15.50	91.50
Lymphocytes (%)	38	42.18	26.04	3.15	88.06
PMN (%)	38	43.20	24.26	6.20	90.32
Macrophages (%)	38	14.32	9.11	2.31	35.66
Leucocytes CD11b^+^ (%)	40	61.80	23.99	16.00	97.00
CD11b PMN (MFI)	38	64.45	11.16	40.92	87.62
CD11b macrophages (MFI)	38	107.53	23.91	75.09	177.47
CD11b leucocytes (MFI)	40	58.99	14.89	27.36	88.47
SCC × 1000 cells/mL	42	501.10	952.70	10.00	5771.00
Milk electrical conductivity (mS/cm)	42	6.11	0.41	5.15	7.15
Milk flow rate (L/min)	42	2.36	0.61	0.91	3.92

**Table 2:** Estimated average value of cell viability, milk leukocyte subsets, CD11b expression and automated recorded farm parameters in POS and NEG groups.

Item	Group ^1^	LS Mean	SE	*p*-Value
Cell viability (%)	POS	56.31	6.25	0.81
	NEG	54.64	3.37	
Lymphocytes (%)	POS	26.26	8.38	0.04
	NEG	45.90	4.59	
PMN (%)	POS	55.22	8.1	0.13
	NEG	40.91	4.44	
Macrophages (%)	POS	18.26	2.96	0.12
	NEG	12.89	1.52	
Leucocytes CD11b^+^ (%)	POS	77.56	7.69	0.03
	NEG	58.31	4.08	
CD11b PMN (MFI ^2^)	POS	67.70	4.08	0.50
	NEG	64.54	2.23	
CD11b macrophages (MFI ^2^)	POS	102.97	7.93	0.52
	NEG	108.80	4.34	
CD11b leucocytes (MFI ^2^)	POS	62.98	5.21	0.11
	NEG	58.87	2.76	
SCC × 1000 cells/mL	POS	1428.14	419.26	0.04
	NEG	448.20	203.42	
Milk electrical conductivity (mS/cm)	POS	6.45	0.14	0.01
	NEG	6.03	0.07	
Milk flow rate (L/min)	POS	1.98	0.19	0.03
	NEG	2.46	0.09	

^1^ Group POS = samples positive to bacteriological analysis (*n* = 9); Group NEG = samples negative to bacteriological analysis (*n* = 34). ^2^ MFI = mean fluorescent intensity used to evaluate the CD11b expression.

**Table 3:** Linear discrimination analysis (LDA): the assignment of individuals (milk samples) to each class (no samples were found in Class A-POS).

Class	A-NEG	B-NEG	B-POS	C-NEG	C-POS	Total
**A-NEG**	13	0	0	0	0	13
	100	0	0	0	0	100
**B-NEG**	1	2	0	2	1	6
	16.67	33.33	0	33.33	16.67	100
**B-POS**	1	0	2	0	1	4
	25	0	50	0	25	100
**C-NEG**	0	0	0	11	0	11
	0	0	0	100	0	100
**C-POS**	0	0	0	0	5	5
	0	0	0	0	100	100
**Total**	15	2	2	13	7	39
	38.46	5.13	5.13	33.33	17.95	100
**Error (%)**	0	0.67	0.50	0	0	0.15
**Prior probability**	0.33	0.15	0.10	0.28	0.13	

**Table 4:** Linear discriminant analyses and weights of the original variable on the canonical variates.

Item	Can1	Can2	Can3	Can4
**Cell viability**	−0.05	0.05	0.01	0.00
**Lymphocytes (%)**	−1.29	−0.70	−0.86	−0.24
**PMN (%)**	−1.54	−0.76	−0.38	−0.44
**Macrophages (%)**	−1.67	−0.75	−0.44	−0.51
**Leukocytes CD11b^+^ (%)**	0.22	0.03	−0.49	0.24
**CD11b PMN (MFI)**	−0.12	0.03	0.03	−0.02
**CD11b macrophages (MFI)**	0.00	0.00	0.02	0.03
**CD11b leukocytes (MFI)**	0.12	0.02	−0.06	0.00
**Milk electrical conductivity (mS/cm)**	−0.72	−0.06	0.18	1.28
**Milk flow rate (L/min)**	0.29	2.21	−0.71	0.42
**Variance**	0.62	0.26	0.09	0.03
**Wilks’ Lambda**	0.11	0.36	0.69	0.91
***p-*** **Value**	0.007	0.272	0.796	0.897

**Table 5:** Mahalanobis distance (*p*-value) among different classes of milk.

Class	A-NEG	B-NEG	B-POS	C-NEG	C-POS
A-NEG	1	0.1741	0.1657	0.0017	0.0012
B-NEG	0.1741	1	0.9438	0.542	0.2541
B-POS	0.1657	0.9438	1	0.3011	0.2834
C-NEG	0.0017	0.542	0.3011	1	0.038
C-POS	0.0012	0.2541	0.2834	0.038	1
